# Modeled exposure to tetrachloroethylene-contaminated drinking water and the occurrence of birth defects: a case-control study from Massachusetts and Rhode Island

**DOI:** 10.1186/s12940-018-0419-5

**Published:** 2018-11-06

**Authors:** Ann Aschengrau, Lisa G. Gallagher, Michael Winter, Lindsey Butler, M. Patricia Fabian, Veronica M. Vieira

**Affiliations:** 1 Department of Epidemiology, Boston University School of Public Health, 715 Albany Street, Talbot 3 East, Boston, MA 02118 USA; 2 Biostatistics and Epidemiology Data Analytics Center, Boston University School of Public Health, 715 Albany Street, Fuller-9, Boston, MA 02118 USA; 3 Department of Environmental Health, Boston University School of Public Health, 715 Albany Street, Talbot 4 West, Boston, MA 02118 USA; 4 University of California, Irvine, Program in Public Health, 653 East Peltason Drive, Irvine, CA 92697 USA

**Keywords:** Tetrachloroethylene, Drinking water, Birth defects

## Abstract

**Background:**

Residents of Massachusetts and Rhode Island were exposed to tetrachloroethylene-contaminated drinking water from 1968 through the early 1990s when it leached from the vinyl lining of asbestos cement water distribution pipes. While occupational exposure to solvents during pregnancy has consistently been linked to an increased risk of certain birth defects, mixed results have been observed for environmental sources of exposure, including contaminated drinking water. The present case-control study was undertaken to examine further the association between prenatal exposure to tetrachloroethylene-contaminated drinking water and the risk of central nervous system defects, oral clefts and hypospadias.

**Methods:**

Cases were comprised of live- and stillborn infants delivered between 1968 and 1995 to mothers who resided in 28 Massachusetts and Rhode Island cities and towns with some PCE-contaminated water supplies. Infants with central nervous system defects (*N* = 268), oral clefts (*N* = 112) and hypospadias (*N* = 94) were included. Controls were randomly selected live-born, non-malformed infants who were delivered during the same period and geographic area as cases (*N* = 771). Vital records and self-administered questionnaires were used to gather identifying information, birth defect diagnoses, and other relevant data. PCE exposure during the first trimester was estimated using water distribution system modeling software that incorporated a leaching and transport model. Prenatal PCE exposure was dichotomized as “high” or “low” exposure at the level corresponding to an estimated average concentration of 40 μg/L, the criterion for remediation when PCE contamination was discovered in 1980.

**Results:**

Mothers with “high” levels of exposure to PCE-contaminated drinking water during the first trimester (> 40 μg/L) had increased odds of having a child with spina bifida (OR: 2.0, 95% CI: 0.8–5.4), cleft lip with or without cleft palate (OR: 3.8, 95% CI: 1.2–12.3) and hypospadias (OR: 2.1, 95% CI:0.5–8.3). No increases in the odds of other defects were observed in relation to “high” exposure levels.

**Conclusions:**

The results of the present study suggest that mothers with “high” PCE exposure levels during the first trimester have increased odds of having a child with spina bifida, cleft lip with or without cleft palate, and hypospadias. These findings support several prior studies that observed an increased risk of selected birth defects following prenatal exposure to solvents in occupational and environmental settings. Even though PCE contamination from vinyl lined pipes was remediated many years ago, it remains a widespread contaminant across the U.S and so environmental regulations must be guided by a precautionary perspective that safeguards pregnant women and their offspring.

## Background

Tetrachloroethylene (PCE) is a commercially important solvent used in dry cleaning, textile processing, and metal degreasing [1]. Because most of its use occurs in poorly controlled workplaces such as dry cleaning businesses and garages, PCE is a frequent contaminant of public drinking water supplies in the United States [2, 3]. The most recent sampling survey from the U.S. Geological Survey found PCE in 7% of surface water samples and 24% of groundwater samples [3].

While improper waste disposal is the typical source of PCE-contaminated drinking water, the public drinking water supplies in New England were affected when PCE leached from the vinyl lining (VL) of asbestos cement (AC) water distribution pipes. The liner, which was applied to the interior of the pipe in a slurry of vinyl toluene resin and PCE, had been introduced in 1968 in response to complaints about water’s taste and odor [4]. Pipes were dried for 48 h before shipping assuming that most of the PCE would evaporate by the time the pipes were installed [5]. Yet, more than a decade lapsed before it was discovered that large quantities of PCE remained in the liner and leached into the public drinking water supplies.

Officials in Massachusetts (MA) and Rhode Island (RI) determined that approximately 750 miles of VLAC pipes had been installed in about 100 cities and towns in the two states [4]. Because the lined pipes were used to replace existing pipe and extend the water system as the population grew, neighboring streets had different contaminant levels.

Levels in MA water monitoring samples taken in 1980 ranged from 1.5 to 80 μg/l in medium and high flow locations and 1600 to 7750 μg/l in low flow locations [5]. RI samples from eleven systems had levels that exceeded the 1980 EPA suggested no adverse response level (SNARL) of 40 μg/L [6]. Remediation to bring water levels below the SNARL was subsequently initiated by bleeding and flushing the pipes [5]. The maximum contaminant level is currently 5 μg/l and reported levels in MA and RI are now below this level [7, 8].

PCE’s neurotoxic and carcinogenic effects have been well-established [1, 9]. There is also evidence of adverse effects on birth outcomes, including congenital anomalies. Animal experiments suggest that prenatal exposure to PCE and trichloroethylene (TCE) increase the risk of anomalies (e.g., [10, 11]). Several epidemiological studies have also reported associations between occupational exposure to organic solvents during pregnancy and congenital anomalies [12–19]; however, the literature examining women with community exposure to contaminated air and drinking water has conflicting findings [20–28].

Our prior retrospective cohort study in Cape Cod, MA found that women with any prenatal PCE exposure had infants with a 3.1-fold increased risk of central nervous system defects (95% CI: 0.9–11.0), a 3.5-fold increased risk of neural tube defects (95% CI: 0.8–14.0), a 3.2-fold increased risk of oral clefts (95% CI: 0.7–15.0), and a 1.4-fold increased risk of hypospadias (95% CI: 0.4–5.4) [20]. Because these findings were based on a small number of cases identified via maternal reports, we undertook present case-control study to examine further the association between PCE-contaminated drinking water and the risk of birth defects with a larger number of cases identified through vital records.

## Methods

### Selection of study population

Cases were comprised of live- and stillborn infants who were delivered from 1968 through 1995 to residents of 24 MA and four RI cities and towns with some VLAC water distribution pipes. Approximately 480 miles of VLAC pipes were installed in these towns, representing approximately 63% of the VLAC pipes in the two states. The remaining RI and MA towns with VLAC pipes were excluded from the present study because they had few VLAC pipes, lacked documentation on the locations and dates of VLAC pipe installation, and/or small resident populations. Available water sampling data indicated that PCE contamination persisted in public water supplies of selected towns through the 1990s because the target level for remediation was 40 μg/L [7].

Cases were identified by abstracting birth defect diagnoses from livebirth, fetal death, and death certificate records (*N* = 479). Based on associations observed in our prior cohort study [20], infants with the following defects were selected: central nervous system defects (CNS) (including anencephaly, spina bifida with or without hydrocephalus, encephalocele, hydrocephalus alone, microcephaly and other CNS defects), oral clefts (cleft lip with and without cleft palate and cleft palate alone), and hypospadias. Cases with more than one birth defect were eligible for inclusion; however, those with recognized syndromes were excluded. Prenatal testing followed by elective termination of affected pregnancies was uncommon during the case ascertainment period [29, 30].

Controls were randomly selected from livebirth records of non-malformed infants born during the same period to residents of the same geographic area as the cases. The control selection process was stratified by state and birth year so that the number of controls selected from MA and RI was proportional to their number of births over the ascertainment period (45% from RI and 55% from MA). A total of 800 controls were targeted for selection; 794 remained after excluding duplicate subjects.

### Questionnaire and vital record data collection

Paper copies of livebirth, fetal death and death certificates and computerized vital record data were abstracted to obtain parent’s and infant’s names; maternal address at delivery; infant’s date of birth; maternal age, race, and educational level; paternal age and educational level; maternal pregnancy history; date of last menstrual period; prenatal care information and birth defect diagnoses.

Mothers were subsequently traced and sent self-administered questionnaires. Overall, 4.2% of case mothers (*N* = 20) and 7.2% of control mothers (*N* = 57) were found to be deceased. We successfully located 87.4 and 88.3% of living case (*N* = 401) and control mothers (*N* = 651), respectively, and, of these, 38.6% of case mothers (*N* = 155) and 31.8% of control mothers (*N* = 207) returned the questionnaire after two mail and one telephone reminder. The purpose of the questionnaire was to identify mothers who moved during pregnancy and augment vital records data on birth defect diagnoses and confounding variables.

When we compared the demographic characteristics of questionnaire respondents and non-respondents, we found that the two groups were similar with respect to PCE exposure status (68.5% of case respondents versus 68.0% of case non-respondents and 66.2% of control respondents versus 70.7% of control non-respondents) and delivery year (e.g., 28.9% of case respondents versus 26.7% of case non-respondents and 37.4% of control respondents versus 37.7% of control non-respondents delivered during 1968–1978). While respondents were more likely to reside in Massachusetts (77.2% of case respondents versus 66.8% of case non-respondents and 72.8% of control respondents versus 51.0% of control non-respondents), be older at delivery (median age was 29.0 for case respondents versus 26.7 for case non-respondents and 28.3 for control respondents versus 26.6 for control non-respondents), and begin prenatal care in the first trimester (93.9% of case respondents versus 85.3% of case non-respondents and 90.8% of control respondents versus 86.3% of control non-respondents), these differences were present for both case and control mothers who returned our study questionnaire.

### Geocoding of residential addresses

Residential addresses at birth recorded in vital records were geocoded to a latitude and longitude using ArcGIS (10.0, ESRI, Redlands, CA). First trimester addresses of questionnaire respondents who moved during pregnancy were also geocoded (*N* = 33 cases and 33 controls). Whenever possible, each address was assigned to a parcel of land. Addresses that could not be geocoded to a parcel were geocoded to the closest parcel address by street number. If a street number was unavailable, the address was geocoded to the middle of the street when the street was less than a mile long or to the intersection of the address with the cross-street when the street was a mile or longer. Overall 98.7% of the addresses were successfully geocoded. All geocoding was conducted without knowledge of case-control status.

### PCE exposure assessment

Because historical water sampling data for PCE was scant, we estimated each subject’s prenatal exposure used a leaching and transport model. The model was developed for our research by Webler and Brown [31, 32] to estimate the mass of PCE entering the drinking water using the starting quantity of PCE in the pipe liner, the pipe’s age, the leaching rate of PCE from the liner into the water, and the water flow rate through the pipe. The initial amount of PCE in the liner was based on the pipe dimensions and information from the manufacturer on the application of the liner. The leaching rate of PCE was determined in experiments by Demond [5].

We incorporated the Webler Brown algorithm into the open source code of EPANET, water distribution system modeling software created by the US EPA [33]. Initially designed to investigate water quality problems, EPANET has been used in several epidemiological studies investigating adverse health effects of drinking water contaminants (e.g. [34]). The combined modeling approach integrated pipe schematics, water use, and PCE transport to determine the water flow rate and direction and the amount of PCE at points of consumption throughout the distribution system.

GIS maps of geocoded residences and the water distribution systems were used to construct a graphic for each town depicting the locations and characteristics of the pipes (e.g. VLAC or not) and consumption points. We assigned each mother’s residence to the closest consumption point on the distribution system. The model simulation estimated the average mass of PCE in grams delivered to each subject’s residence during the calendar year when the first trimester ended. We estimated the annual exposure because we did not have data on the month of move-in or VLAC pipe installation. Average monthly exposure was obtained by dividing annual exposures by 12. Questionnaire respondents who report using a private well for their prenatal water supply (*N* = 21) were considered unexposed.

### Data analysis

The strength of the association between PCE exposure and each birth defect was estimated with odds ratios (OR) and statistical stability was evaluated with 95% confidence intervals. The following defect groups were examined: any central nervous system defect, any neural tube defect, any anencephaly, any spina bifida (with and without hydrocephalus), any other CNS defects, any oral cleft, any cleft lip (with or without cleft palate), cleft palate alone, and hypospadias. Cases with more than one of these defects contributed to each subgroup. Analyses of hypospadias were limited to male cases and controls. ORs were calculated only if there were at least three exposed cases and three exposed controls.

Analyses first compared mothers who were ever exposed to PCE-contaminated drinking water during the calendar year the first trimester ended to unexposed mothers. We focused on exposure during the first trimester because the structural defects under study form during this period [35]. Next, we dichotomized PCE exposure at 1.136 g, the level corresponding to an average monthly drinking water concentration of 40 μg/L during the calendar year that the first trimester ended. This concentration was the criterion for remediation when PCE contamination was discovered in 1980. Levels above 40 μg/L were designated as “high.” Each set of analyses was repeated after (1) incorporating exposure levels at the first trimester address for questionnaire respondents who moved during pregnancy, (2) excluding cases and controls with a family history of defects, (3) excluding cases with multiple defects, and (4) excluding cases included in our prior cohort study (*N* = 10).

Logistic regression models estimated ORs while controlling for confounding variables. We selected potential confounders from those available in the vital records and self-administered questionnaires based on a literature review and construction of directed acyclic graphs. Multiple imputation was used to obtain values of potential confounders with missing data. The amount of missing data ranged from 0% (state of residence and delivery year) to 72.5% (maternal alcoholic beverage consumption). Variables with a high proportion of missing data came solely from the self-administered questionnaires. Twenty imputed data sets were generated using the fully conditional specification (FCS) multiple imputation method based on 27 variables. Point and variance estimates from the imputed data sets were subsequently combined and used in adjusted analyses.

Initial adjusted models controlled for one potential confounder at a time. Controlling for delivery year had the greatest impact on the crude associations and so it was included in all multivariate models. We also decided to include state in all multivariable models to account for variations in case ascertainment between the two states. Additional variables that changed the crude association between PCE exposure and each type of birth defect by > = 10% were also included in the final models; these included maternal alcoholic beverage consumption in the oral cleft analyses and maternal race in the hypospadias analyses.

## Results

The final analysis was based on 268 cases with CNS malformations (including 151 neural tube defects and 117 other CNS defects), 112 cases with oral clefts (including 59 with cleft lip with or without cleft palate and 53 with cleft palate alone), 94 cases with hypospadias, and 771 controls. These numbers include 10 cases identified through the maternal questionnaire: 7 cases of hypospadias, 1 case of spina bifida and 2 cases of cleft lip with cleft palate. In addition, these numbers exclude 5 cases and 11 controls whose residential information was insufficient for geocoding, 12 controls whose questionnaire data indicated the presence of a congenital anomaly (that was not recorded in state records), and 12 cases whose questionnaire data indicated that the defect was part of a syndrome (also omitted from state records).

Overall, 68.3% of CNS cases, 72.3% of oral cleft cases, 61.7% of hypospadias cases and 67.8% of control mothers had some PCE exposure during the first trimester. The exposure distributions of cases and controls spanned several orders of magnitude (Table 1). There were no discernable trends in the exposure distribution of cases versus controls, except at the upper end of the distribution where higher exposure levels were observed among cases.

**Table 1:** Distribution of Average Monthly PCE Exposure (g) During Year First Trimester Ended Among All Exposed Case and Control Mothers

	Exposed	Exposed
	Case Mothers	Control Mothers
Minimum	5.2 × 10^− 7^	2.1 × 10^− 8^
5th Percentile	2.1 × 10^− 5^	2.2 × 10^− 5^
10th Percentile	7.7 × 10^− 5^	5.4 × 10^− 5^
25th Percentile	4.4 × 10^− 4^	6.8 × 10^− 4^
Median	3.4 × 10^− 3^	5.9 × 10^− 3^
75th Percentile	3.9 × 10^− 2^	5.6 × 10^− 2^
90th Percentile	5.0 × 10^− 1^	4.3 × 10^− 1^
95th Percentile	1.8	1.2
Maximum	24.8	81.81

Both case and control mothers were predominantly white but, depending on the case group, differed on many other demographic and behavioral characteristics, such as state of birth, delivery year, maternal educational level and prenatal cigarette smoking (Table 2).

**Table 2:** Distribution of selected characteristics of cases and controls

Characteristic	CNS Cases (*N* = 268)		Oral Clef Cases (*N* = 112)		Hypospadias Cases (*N* = 94)		All Controls (*N* = 771)	
	n	%	n	%	n	%	n	%
Maternal residence at delivery								
Massachusetts	167	62.3	85	75.9	81	86.2	436	56.5
Rhode Island	101	37.7	27	24.1	13	13.8	335	43.5
Infant year of birth								
1969–1978	98	36.6	20	17.9	14	14.9	275	35.7
1979–1988	88	32.8	45	40.2	27	28.7	290	37.6
1989–1995	82	30.6	47	42.0	53	56.4	206	26.7
Infant gender								
Male	131	49.4	65	58.0	94	100	408	52.9
Female	134	50.6	47	42.0	–	–	363	47.1
Missing	3							
Maternal age at delivery (mean, sd)	26.3 (6.1)		27.8 (5.3)		29.3 (5.2)		27.0 (5.4)	
Missing	3		0		0		0	
Paternal age at delivery (mean, sd)	29.3 (6.3)		30.8 (5.8)		31.7 (6.2)		29.8 (6.1)	
Missing	30		11		0		34	
Maternal race								
White	201	90.5	94	92.2	86	96.6	613	90.1
Non-White	21	9.5	8	7.8	3	3.4	67	9.9
Missing	46		10		5		91	
Maternal educational level								
< High school	36	16.7	10	9.9	7	7.9	96	14.3
High school graduate	104	48.1	42	41.6	26	29.2	244	36.4
Some college	36	16.7	32	31.7	26	29.2	172	25.7
College graduate	40	18.5	17	16.8	30	33.7	158	23.6
Missing	52		11		5		101	
Prenatal care began in first trimester								
Yes	162	84.4	89	89.9	85	97.7	473	87.9
No	30	15.6	10	10.1	2	2.2	65	12.1
Missing	76		13		7		233	
Prenatal cigarette smoking								
Yes	29	21.8	21	31.8	12	15.6	70	24.3
No	104	78.2	45	68.2	65	84.4	218	75.7
Missing	135		46		17			483
Prenatal alcoholic beverage consumption								
Yes	19	23.8	19	51.4	9	29.0	69	35.6
No	61	76.3	18	48.6	22	71.0	125	64.4
Missing	188		75		63		577	
Number of prior livebirths								
0	91	41.4	38	37.3	43	47.8	296	43.8
1	68	30.9	36	35.3	28	31.1	217	32.1
2+	61	27.7	28	27.5	19	21.1	163	24.1
Missing	48		10		4		95	
Possible maternal exposure to solvents in workplace^a^								
Yes	2	2.5	5	13.9	1	3.1	4	2.1
No	79	97.5	31	86.1	31	96.9	185	97.9
Missing	187		76		62		582	

^a^Ascertained from a series of questions on jobs and job activities that typically involve solvent exposure (e.g., metal degreasing)

Any PCE exposure during the first trimester was not associated with meaningful increases in the adjusted odds of all CNS defects combined, neural tube defects, anencephaly, spina bifida, other CNS defect or hypospadias (Table 3). In contrast, any first trimester PCE exposure was associated with a 2.1-fold increase in the adjusted odds of cleft lip with or without cleft palate (95% CI: 1.1–4.0) that rose to 3.8 for prenatal PCE exposure levels above 40μg/L (95% CI: 1.2–12.3) (*p* value for trend 0.13). “High” PCE exposure levels were also associated with increases in the adjusted odds of spina bifida with and without hydrocephalus (OR: 2.0, 95% CI: 0.8–5.4) and hypospadias (OR: 2.1, 95% CI: 0.5–8.3). No increases in the odds of other CNS defects were observed in relation to “high” PCE exposure levels. Only one case of cleft palate alone was exposed to “high” PCE exposure levels and so we did not estimate its associated odds ratio; any PCE exposure was associated with a 1.4-fold increase in the adjusted odds of cleft palate alone (95% CI: 0.7–2.7).

**Table 3:** Frequencies, Odds Ratios and 95% Confidence Intervals for Birth Defects According to Average Monthly PCE Exposure during Year First Trimester Ended

	Number of Cases	Number of Controls	Crude Odds Ratio (95% CI)	Multivariable^a^ Odds Ratio (95% CI)
All Central Nervous System Defects (*N* = 268)				
Any Exposure	183	523	1.0 (0.8–1.4)	1.1 (0.8–1.6)
No Exposure	85	248	1.0 (−-----)	
Exposure Categorized by 1980 Action Level				
> 1.136 g (>40μg/L)	13	32	1.2 (0.6–2.4)	1.2 (0.6–2.4)
> 0- < =1.136 g	170	491	1.0 (0.7–1.4)	1.1 (0.8–1.6)
0 (Referent)	85	248	1.0 (−-----)	
*P* value for trend				0.64
All Neural Tube Defects (*N* = 151)				
Any Exposure	100	523	0.9 (0.6–1.3)	1.1 (0.8–1.7)
No Exposure	51	248	1.0 (−-----)	
Exposure Categorized by 1980 Action Level				
> 1.136 g (>40μg/L)	11	32	1.7 (0.8–3.5)	1.5 (0.7–3.1)
> 0- < =1.136 g	89	491	0.9 (0.6–1.3)	1.1 (0.7–1.7)
0 (Referent)	51	248	1.0 (−-----)	
*P* value for trend				0.33
Anencephaly (*N* = 82)				
Any Exposure	54	523	0.9 (0.6–1.5)	1.1 (0.7–1.9)
No Exposure	28	248	1.0 (−-----)	
Exposure Categorized by 1980 Action Level				
> 1.136 g (>40μg/L)	5	32	1.4 (0.5–3.8)	1.1 (0.4–3.1)
> 0- < =1.136 g	49	491	0.9 (0.5–1.4)	1.1 (0.6–1.9)
0 (Referent)	28	248	1.0 (−-----)	
*P* value for trend				0.86
Spina bifida with and without hydrocephalus (*N* = 69)				
Any Exposure	46	523	0.9 (0.6–1.6)	1.2 (0.7–2.2)
No Exposure	23	248	1.0 (−-----)	
Exposure Categorized by 1980 Action Level				
> 1.136 g (>40μg/L)	6	32	2.0 (0.8–5.3)	2.0 (0.8–5.4)
> 0- < =1.136 g	40	491	0.9 (0.5–1.5)	1.1 (0.6–2.1)
0 (Referent)	23	248	1.0 (−-----)	
*P* value for trend				0.16
Other CNS Defects (*N* = 117)				
Any Exposure	83	523	1.2 (0.8–1.8)	1.1 (0.7–1.8)
No Exposure	34	248	1.0 (−------)	
Exposure Categorized by 1980 Action Level				
> 1.136 g (>40μg/L)	2	32	------^b^	------^b^
> 0- < =1.136 g	81	491	1.2 (0.8–1.8)	1.2 (0.7–1.9)
0 (Referent)	34	248	1.0 (−-----)	
*P* value for trend				N/A
All Oral Clefts (*N* = 112)				
Any Exposure	81	523	1.2 (0.8–1.9)	1.7 (1.0–2.8)
No Exposure	31	248	1.0 (−-----)	
Exposure Categorized by 1980 Action Level				
> 1.136 g (> 40μg/L)	6	32	1.5 (0.6–3.9)	2.2 (0.8–6.0)
> 0- < =1.136 g	75	491	1.2 (0.8–1.9)	1.7 (1.0–2.8)
0 (Referent)	31	248	1.0 (−-----)	
*P* value for trend				0.13
Cleft Lip with or without Cleft Palate (*N* = 59)				
Any Exposure	44	523	1.4 (0.8–2.5)	2.1 (1.1–4.0
No Exposure	15	248	1.0 (−-----)	
Exposure Categorized by 1980 Action Level				
> 1.136 g (>40μg/L)	5	32	2.6 (0.9–7.6)	3.8 (1.2–12.3)
> 0- < =1.136 g	39	491	1.3 (0.7–2.4)	1.9 (1.0–3.8)
0 (Referent)	15	248	1.0 (−-----)	
*P* value for trend				0.02
Cleft Palate Alone (*N* = 53)				
Any Exposure	37	523	1.1 (0.6–2.0)	1.4 (0.7–2.7)
No Exposure	16	248	1.0 (−-----)	
Exposure Categorized by 1980 Action Level				
> 1.136 g (>40μg/L)	1	32	------^b^	------^b^
> 0- < =1.136 g	36	491	1.1 (0.6–2.1)	1.4 (0.7–2.8)
0 (Referent)	16	248	1.0 (−-----)	
*P* value for trend				N/A
Hypospadias (*N* = 94)^c^				
Any Exposure	58	266	0.9 (0.5–1.4)	1.3 (0.8–2.2)
No Exposure	36	142	1.0 (−-----)	
Exposure Categorized by 1980 Action Level				
> 1.136 g (>40μg/L)	3	11	1.1 (0.3–4.1)	2.1 (0.5–8.3)
> 0- < =1.136 g	55	255	0.9 (0.5–1.4)	1.3 (0.8–2.2)
0 (Referent)	36	142	1.0 (−-----)	
*P* value for trend				0.31

^a^All adjusted analyses control for state of birth, delivery year. In addition, maternal alcohol use during pregnancy is controlled in the oral cleft analysis and maternal race is controlled in the hypospadias analysis

^b^Not calculated

^c^Analysis includes only male cases and controls

N/A not applicable

No meaningful changes in these results were observed when the analysis incorporated exposure levels at the first trimester address for questionnaire respondents who moved during pregnancy or excluded cases and controls with a family history of defects, cases with multiple defects, or cases in our prior cohort study (data not shown).

## Discussion

The results of the present study suggest that pregnant women exposed to “high” levels of PCE-contaminated drinking water during the first trimester have an increased odds of spina bifida, cleft lip with or without cleft palate and hypospadias. No evidence of increases in the odds of other defects were observed in relation to “high” exposure levels.

Several limitations should be considered when interpreting these results. First, these findings are statistically unstable and have wide confidence intervals because a small number of mothers were exposed to PCE levels above the remediation cut point. Second, even though results from a prior validation study indicate good correlation between our modeled exposure estimates and PCE concentrations in historical water samples from Cape Cod (Spearman correlation coefficient (ρ) = 0.65, *p* < 0.00010 [36], non-differential exposure misclassification stemming from our model-based exposure estimates likely attenuated the observed associations. In addition, our leaching and transport model did not estimate personal exposure levels but rather the mass of PCE delivered to the home. Behavioral data on prenatal water consumption and bathing habits during 1969–1995 were available only for questionnaire respondents and considered poor quality because of the long recall period. Thus, it was not incorporated into our analysis.

Still another limitation arose from using vital records to identify birth defect cases and non-malformed controls because this source of information was likely to suffer from mis-reporting and under-reporting. Direct evidence for these inaccuracies comes from the questionnaire data where mothers reported 12 syndromes that had been recorded as single defects in vital records and 22 defect diagnoses that were not recorded in vital records. Indirect evidence comes from birth defects surveillance data in Massachusetts and Rhode Island that suggest the number of cases identified (with the possible exception of anencephaly) was lower than expected when multiple sources of case ascertainment are used [37, 38]. However, even though there was a great deal of publicity when the contamination was discovered in 1980, possible carcinogenic effects were the focus of concern in newspaper reports (e.g., [39]), and so there is no reason to believe that recording of defects in vital records was related to PCE exposure. There is also no reason to believe that reporting of defects on the questionnaire was related to PCE exposure since only 2% of exposed mothers thought that their drinking water was contaminated with PCE. Thus, these errors were likely non-differential thereby biasing the results towards the null.

Because VLAC pipes were used to replace existing pipes and expand the distribution system in response to the growing population, the geographic pattern of contamination was irregular. In fact, depending on the direction of water flow, neighboring homes could have very different exposure levels. Thus, despite several different characteristics among cases and controls, little confounding was observed in the analysis likely from the lack of associations between PCE exposure and potential confounders. Multiple imputation procedures were used fill in missing values on confounders. While they are considered the most valid approach for handing this problem [40], we cannot rule out the possibility that residual confounding affected the results.

Selection bias was unlikely because we were unaware of the exposure status when we selected cases and controls for study. Despite the low questionnaire response rate and the numerous demographic differences between respondents and non-respondents, these differences were present for both cases and controls and so were unlikely sources of bias. Moreover, analyses were conducted on the entire sample, irrespective of respondent status.

Because we investigated defect diagnoses at birth, it is possible that a differential loss of pregnancies before this point may have led us to underestimate the true effect of PCE exposure. Such a ‘competing mortality’ bias could exist if the pregnancies most susceptible to PCE-induced birth defects were not included in our study because they were more likely to be spontaneously aborted earlier in pregnancy. Previous findings from our prior research, however, suggest that prenatal PCE exposure is not associated with early pregnancy loss [41].

Numerous animal experiments have found an adverse effect of prenatal exposure to PCE, the closely related solvent trichloroethylene (TCE), and their metabolite trichloroacetic acid (TCA) on the risk of congenital defects. An increased prevalence of cardiovascular, musculoskeletal, central nervous system and ocular anomalies have been observed among chicks and rats over a wide range of prenatal exposure levels (e.g., [10, 11]).

Many epidemiological studies have also found positive associations between occupational exposure to organic solvents during pregnancy and the risk of congenital anomalies, including oral clefts, neural tube defects and cardiac defects [12–14, 18, 19, 42]. However, these studies are difficult to interpret because broad exposure categories including many different solvents were typically examined. Studies of dry cleaning workers with only PCE exposure were too small to provide meaningful results [43–46].

A smaller body of literature examining women exposed via contaminated air and drinking water has less consistent results with several studies reporting increases in the risk of birth defects associated with PCE and/or TCE exposure [20–26] while others reporting null findings [24, 27, 28].

Differing results have also been observed for drinking water studies that examined the defects included in our investigation. A 1986 study of well water contaminated with TCE (267 ppb), PCE (21 ppb), and other compounds in Woburn, MA found an increased prevalence of central nervous system, oral cleft and chromosomal defects combined among children with prenatal exposure [23]. While a follow-up study also found that Woburn residents had a higher prevalence of certain defects, including neural tube defects, cleft lip, hypospadias and chonal atresia [27, 47], investigators attributed this to differences in case ascertainment between Woburn and the comparison population [27]. The similar frequency of defects before and after the affected wells were closed also led investigators to conclude that their occurrence was not associated with prenatal exposure to contaminated well water.

In contrast, a 1995 New Jersey study found that PCE drinking water levels > 10 ppb were associated with a 3.5-fold increased risk of oral clefts (90% CI: 1.3–8.8), TCE drinking water levels > 5 ppb were associated with a 2.2 fold increased risk of oral clefts (90% CI: 1.2–4.2), and TCE levels > 10 ppb were associated with a 2.5-fold increased risk of neural tube defects (90% CI: 0.9–6.4) [22]. Positive associations were also observed between any prenatal exposure to PCE-contaminated drinking water and the risk of neural tube defects (OR: 3.2, 95% CI: 0.9–11.0), and oral clefts (OR: 3.0, 95% CI: 0.7–13.0), and hypospadias (OR: 1.4, 95% CI: 0.4–5.4) in our previous study of births from Cape Cod MA [20]. Lastly, a study from Marine Corps Base Camp Lejeune found a 2.4-fold increased odds of neural tube defects in relation to TCE exposure levels > 5 ppb (95% CI: 0.6–9.6) but no associations between neural tube defects and oral clefts and any level of PCE exposure [24].

The two studies using different metrics for airborne solvent exposure also have conflicting results for the defects examined in the present investigation. A study using broadly categorized census-tract estimates of TCE levels reported a 2.0-fold increased odds of spina bifida for medium exposure levels (0.5–.16 μg/m^3^) (95% CI: 1.1–3.6) and a 1.3-fold increased odds for high exposure levels (> 0.16 μg/m^3^) (95% CI: 0.6–2.8) [25] while a study using a highly refined emission weighted residential proximity model found no associations between PCE and TCE and either neural tube defects or oral clefts (PCE ORs and 95% CI: 0.92, 0.81–1.06 and 0.98, 0.88–1.08, respectively, and TCE ORs and 95% CI: 0.95, 0.83–1.09 and 1.02, 0.92–1.13, respectively) [28].

Possible explanations for differing results among the environmental studies include small numbers of defect cases leading to unstable results, exposure misclassification, inadequate control of confounding, as well as differences in exposure assessment methods and exposure levels. In particular, it is possible that the stronger associations observed in our prior cohort study in Cape Cod MA resulted from higher exposure levels. The average monthly exposure levels in our prior cohort study ranged from 9.6 × 10^− 05^ to 131.8 g and the 25th, 50th, 75th and 90th percentiles were 0.1, 0.6, 2.3 and 6.4 g, respectively [20]; see Table 1 for comparison.

In summary, the results of the present study suggest that pregnant women with “high” levels of exposure to PCE-contaminated drinking water (> 40 μg/L) have increased odds of having a child with spina bifida, cleft lip and hypospadias. These findings are biologically plausible given evidence of placental transfer of PCE [48]. They also support the results of several other studies that have observed increased risks of certain birth defects following prenatal exposure to solvents in occupational and environmental settings. Even though PCE contamination from VLAC pipes was remediated many years ago and the levels are no longer elevated in MA and RI [7, 8], the solvent remains a common contaminant of U.S. drinking water supplies [1] and so environmental regulations must be guided by a precautionary perspective that safeguards pregnant women and their offspring.

## Abbreviations

CI: Confidence Interval
GIS: Geographic Information System
MA: Massachusetts
OR: Odds Ratio
PCE: Tetrachloroethylene
RI: Rhode Island
SNARL: Suggested no adverse response action level
TCE: Trichloroethylene
VLAC: Vinyl-lined asbestos-cement
